# SARS-CoV-2 Testing of Emergency Department Patients Using cobas^®^ Liat^®^ and eazyplex^®^ Rapid Molecular Assays

**DOI:** 10.3390/diagnostics13132245

**Published:** 2023-07-01

**Authors:** Renate Egerer, Birgit Edel, Franziska Hornung, Stefanie Deinhardt-Emmer, Michael Baier, Jan-Christoph Lewejohann, Wolfgang Pfister, Bettina Löffler, Jürgen Rödel

**Affiliations:** 1 Institute of Medical Microbiology, Jena University Hospital, Friedrich Schiller University, 07747 Jena, Germany; renate.egerer@med.uni-jena.de (R.E.); birgit.edel@med.uni-jena.de (B.E.); franziska.hornung@med.uni-jena.de (F.H.); stefanie.deinhardt-emmer@med.uni-jena.de (S.D.-E.); michael.baier@med.uni-jena.de (M.B.); bettina.loeffler@med.uni-jena.de (B.L.); 2 Department of Emergency Medicine, Jena University Hospital, Friedrich Schiller University, 07747 Jena, Germany; jan-christoph.lewejohann@med.uni-jena.de; 3 Department of Hospital Hygiene, Sophien- und Hufeland-Klinikum, 99425 Weimar, Germany; w.pfister@klinikum-weimar.de

**Keywords:** SARS-CoV-2, POC, emergency department, rapid diagnostic test

## Abstract

Rapid testing for Severe Acute Respiratory Syndrome Coronavirus-2 (SARS-CoV-2) of patients presenting to emergency departments (EDs) facilitates the decision for isolation on admission to hospital wards. Differences in the sensitivity of molecular assays have implications for diagnostic workflows. This study evaluated the performance of the cobas^®^ Liat^®^ RT-PCR, which is routinely used as the initial test for ED patients in our hospitals, compared with the eazyplex^®^ RT-LAMP. A total of 378 oropharyngeal and nasal swabs with positive Liat^®^ results were analysed. Residual sample aliquots were tested using NeuMoDx™, cobas^®^ RT-PCR, and the eazyplex^®^ assay. Patients were divided into asymptomatic (n = 157) and symptomatic (n = 221) groups according to the WHO case definition. Overall, 14% of positive Liat^®^ results were not confirmed by RT-PCR. These samples were mainly attributed to 26.8% of asymptomatic patients, compared to 3.8% of the symptomatic group. Therefore, positive Liat^®^ results were used to provisionally isolate patients in the ED until RT-PCR results were available. The eazyplex^®^ assay identified 62% and 90.6% of RT-PCR-confirmed cases in asymptomatic and symptomatic patients, respectively. False-negative eazyplex^®^ results were associated with RT-PCR Ct values > 30, and were more frequent in the asymptomatic group than in the symptomatic group (38.1% vs. 5.1%, respectively). Both the Liat^®^ and eazyplex^®^ assays are suitable for testing symptomatic patients. Their use in screening asymptomatic patients depends on the need to exclude any infection or identify those at high risk of transmission.

## 1. Introduction

Testing patients for Severe Acute Respiratory Syndrome Coronavirus-2 (SARS-CoV-2) infection, regardless of the presence of typical symptoms, is often performed in the emergency department (ED) prior to their hospital admission [1]. Pending test results, patients are kept in the ED before being transferred to a ward. During epidemic waves this places an enormous strain on ED capacities, and if SARS-CoV-2 diagnosis takes several hours, it can lead to delays in appropriate treatment for critically ill patients [2]. Real-time RT-PCR, conducted using extracted RNA, is considered the gold standard in diagnostics because it combines high sensitivity and specificity. Most RT-PCR assays require an average of 2 to 4 h for test results to be available. This can impede timely patient management in the ED when broad screening of all patients is performed during periods of high incidences in the population [1]. If the molecular diagnostic laboratory is not close to the hospital, transport time and logistics further increase the time to diagnostic reporting. Rapid point-of-care (POC) RT-PCR and isothermal amplification systems with minimal hands-on time are more suitable for timely diagnosis, but the associated cost of consumables is higher and a single instrument does not allow for high sample throughput [3,4,5]. Compared to RT-PCR, isothermal amplification assays are less sensitive [6,7,8,9]. This can be a major drawback, despite their robustness and ease of use. However, analytical sensitivity does not necessarily correspond to clinical relevance [10,11]. Weakly positive RT-PCR results with high Ct values in samples from asymptomatic individuals can lead to unnecessary delays in therapy and excessive repeat testing. Isothermal amplification assays may therefore be a viable alternative for use in clinical settings, where timely identification of acute infections and infectious patients are required [12,13,14].

In our hospitals, the cobas^®^ Liat^®^ system (Roche, Penzberg, Germany), a small instrument for automated RT-PCR testing with a short turnaround time of less than 30 min, has been selected as the primary SARS-CoV-2 screening tool for ED patients [15]. However, due to a significant number of false-positive results during assay validation, it was determined that any positive Liat^®^ result has to be confirmed via RT-PCR for the final diagnostic report, in accordance with the 2021 FDA communication [16,17]. During the 2021/2022 winter epidemic, the diagnostic workflow for samples from the ED consisted of an initial Liat^®^ test with immediate reporting of positive results as preliminary suspect cases to be confirmed as soon as possible using RT-PCR systems. The eazyplex^®^ SARS-CoV-2 RT-LAMP assay (Amplex Diagnostics, Gars-Bahnhof, Germany), a rapid RNA extraction-free isothermal amplification system that provides results within 30 min, was routinely used as a back-up diagnostic when other assays were in short supply.

The aim of this study was to evaluate the utility of the eazyplex^®^ RT-LAMP in comparison to the cobas^®^ Liat^®^ for rapid testing of samples from ED patients with and without symptoms characteristic of acute SARS-CoV-2 infection. Comparative testing was performed on the same swab specimens, and only Copan UTM swabs were used.

## 2. Materials and Methods

### 2.1. Clinical Specimens and Diagnostic Workflow

The samples were oropharyngeal and nasal swabs collected in universal transport medium (UTM-RT MINI swabs, 1 mL, 359C, Copan, distributed by Mast Diagnostica, Reinfeld, Germany) at the ED of the Jena University Hospital and a regional hospital (SHK Weimar) between November 2021 and March 2022.

Samples from the Jena University Hospital were tested using the SARS-CoV-2 cobas^®^ Liat^®^ screening assay 24/7 in the microbiology and clinical chemistry laboratory, which is connected to the ED by a pneumatic tube system for rapid sample transfer. Samples from the external hospital in Weimar were tested at the small on-site laboratory using Liat^®^, and positive specimens were transported to the microbiology laboratory twice a day for confirmatory RT-PCR. Prior to testing, samples were mixed 1:1 with phosphate-buffered saline (PBS, Gibco, Thermo Fisher Scientific, Wesel, Germany). Positive Liat^®^ samples were analysed using RT-PCR during the normal working day, between 7 a.m. and 8 p.m. We used two different RT-PCR systems, the cobas^®^ Roche and the NeuMoDx™ (Qiagen, Hilden, Germany), depending on the availability of test kits and technical problems with the instruments. For this study, residual aliquots of all Liat^®^ positive samples were tested using the eazyplex^®^ RT-LAMP assay within 24 h. Samples were stored at 8 °C until all assays were performed.

### 2.2. SARS-CoV-2 Assays

The cobas^®^ Liat^®^ system is a small device for single-use cartridges with fully automated processing and amplification. For detection of SARS-CoV-2, it utilized a dual target assay; a positive result was reported without releasing Ct values if either or both ORF1 and N target genes were detected. The test run time was 20 min. A total of 200 μL of UTM/PBS was loaded into the cartridge.

For the NeuMoDx™ RT-PCR, 700 μL of the sample was loaded onto the NeuMoDx™ 96 Molecular system. The NeuMoDx™ SARS-CoV-2 assay targeted N and Nsp2 genes. For the cobas^®^ RT-PCR, 600 μL of the sample was loaded onto the cobas^®^ 6800 instrument. The assay used primers for E and ORF1ab genes. All RT-PCR assays were performed according to the manufacturers’ protocols.

For the eazyplex^®^ RT-LAMP assay, 25 μL of UTM/PBS was heated at 99 °C for 2 min before being pipetted into ready-to-use tubes containing 500 μL of resuspension and lysis fluid (RALF). Subsequent testing was performed using a Genie HT instrument (Amplex Diagnostics) according to the manufacturer’s protocol. The assay’s total runtime was 25 min, but a positive result was reported in real time if the fluorescence level of either or both of the N and ORF8 target genes rose above the threshold.

### 2.3. Data Analysis

Medical records were reviewed to group patients as asymptomatic or symptomatic according to the WHO clinical criteria for COVID-19 cases [18]. Patients were classified as symptomatic if they presented with acute onset of fever and cough or with three or more of the following symptoms: fever, cough, weakness/fatigue, headache, myalgia, sore throat, coryza, dyspnoea, and nausea/diarrhea/anorexia. The performance of cobas^®^ Liat^®^ and eazyplex^®^ assays was assessed by calculating the positive percent agreement (PPA) with NeuMoDx™ or cobas^®^ RT-PCR defined as reference. The turnaround times of different assays were calculated using the times recorded in the laboratory information system when tests were requested and results were released.

## 3. Results

In total, 325 of 378 positive Liat^®^ tests were confirmed via RT-PCR (86%). There were only three Liat^®^-positive samples that were negative using RT-PCR, but positive when subsequently testing using the eazyplex^®^ RT-LAMP assay (0.8%). Positive Liat^®^ results that could not be confirmed using either or both of the RT-PCR and RT-LAMP methods were defined as false-positive Liat^®^ results. The false-positive rate of the Liat^®^ assay was high, at 26.8% in the asymptomatic patient group, while only 3.8% of positive Liat^®^ results in symptomatic patients could not be verified (Table 1). The patient medical records of 25% of those with false-positive Liat^®^ test results contained the information that they had been diagnosed with SARS-CoV-2 infection ≥ 2 weeks previously, indicating that the Liat^®^ assay could produce false-positive results relative to the reference method and the absence of symptoms in the patient, but might detect residual amounts of viral RNA from a previous infection.

In this study, the overall sensitivity (PPA) of eazyplex^®^ was only 62% in asymptomatic patients, but 90.9% in symptomatic patients (Table 2). As RT-PCR Ct values ≥ 30 indicate a low risk of viral shedding, we divided the samples into three groups of high (Ct ≤ 25), intermediate (25 < Ct < 30), and low (Ct ≥ 30) viral load to further evaluate the performance of RT-LAMP compared to RT-PCR (Table 3) [10,19]. Not surprisingly, at high viral loads the sensitivity of the eazyplex^®^ reached 95.2% and 100% in asymptomatic and symptomatic patient groups, respectively (Table 3). Samples with intermediate viral loads were detected with sensitivities > 80%. In samples with low viral loads, sensitivity decreased to approximately 10%. It is noteworthy that only 5.2% of symptomatic patients, but 38.1% of asymptomatic patients, had a low viral load (Table 3).

For quality assessment, we analysed reference standards of the Delta and Omicron variants (INSTAND e.V.) containing approximately 10^5^ virus copies/mL, which represented a lower limit of infectivity [20]. As shown in Table 4, the corresponding Ct values for the NeuMoDx™ and cobas^®^ RT-PCR assays were similar, ranging from 27 to 29. Both standards were also detected via RT-LAMP, with a positive result for at least the N gene.

Liat^®^ results were reported to the ED at a median time of 1.1 h (IQR 0.88–1.42, n = 315) after test requests. Confirmatory RT-PCR testing of positive specimens resulted in a median delay of 2.5 h (IQR 1.88–6.33) for the final diagnostic report. For Liat^®^-positive specimens sent from the external hospital to the laboratory for confirmation, the median time from test request to final report was 9 h (IQR 6.25–19.12, n = 63). In the small number of cases in which the eazyplex^®^ was used as a screening assay in the routine workflow, results were available in a median time of 0.9 h (IQR 0.75–1.25, n = 11).

## 4. Discussion

Rapid molecular testing for SARS-CoV-2 in the ED is critical for timely and appropriate decisions regarding further management and isolation of patients [1,5]. RT-PCR tests are the gold standard for reliable identification of SARS-CoV-2 in symptomatic patients due to their high sensitivity. On the other hand, there are strong arguments that low-positive RT-PCR results in patients without characteristic symptoms are not relevant, either for patient management or for identification of infectivity [10,19]. In this context, it should be noted that widespread testing of asymptomatic individuals is expensive, time-consuming, labor intensive, and generates significant amounts of waste [8].

The cobas^®^ Liat^®^, a sensitive RT-PCR designed for use as a POC test, produced a high rate of positive results in asymptomatic patients that could not be confirmed via reference RT-PCR, consistent with data from previous reports [17,21]. It should be noted that this assay was originally intended for use in symptomatic patient testing [17]. Most of the false-positive results were apparently due to the detection of residual nucleic acid from previous infections. The superior sensitivity of RT-PCR, combined with high-speed amplification of short sequences, may increase the risk of detecting residual fragments due to slow degradation of viral RNA, as shown for SARS-CoV-2, influenza virus, and others [10,22]. The need to confirm a positive Liat^®^ result not only adds diagnostic cost, but also delays adequate treatment and care of patients, as each case must be managed as a presumptive COVID-19 patient until the final standard RT-PCR result is available.

The eazyplex^®^ RT-LAMP assay was less sensitive than the Liat^®^, and reached an overall sensitivity ≥ 90% only for symptomatic (but not for asymptomatic) ED patients. However, the usefulness of a rapid diagnostic test in practice can be assessed differently depending on the corresponding RT-PCR Ct values. There is little doubt that a low positive RT-PCR result does not indicate that a patient is infectious, but viral load cut-offs that accurately discriminate whether or not an individual is producing enough virus for transmission are difficult to define, and Ct values can vary between different assays [19,23]. Interestingly, many studies that have examined the relationship between Ct values and the presence of culturable amounts of virus as a marker of sample infectivity have found similar results [10]. Most studies have reported that Ct values < 30 (or even less) are required for successful growth of the virus from samples in cell culture. It has been calculated that one PFU of SARS-CoV-2 corresponds to viral copy numbers between 10^4^ and 10^5^ [24]. These findings are in good agreement with the detection limits determined for the eazyplex^®^ RT-LAMP assay, and are also consistent with the Abbott ID NOW™ isothermal amplification assay, for which high sensitivity has been reported for samples with Ct values < 30 [5,6]. As shown in several studies, the sensitivity of most rapid antigen tests is significantly lower, in the range of 10^6^ to 10^7^ virus copies/mL [25]. Therefore, isothermal amplification assays developed for use with crude samples may provide an alternative tool for initial screening of patients when rapid results are needed.

In principle, both the Liat^®^ and eazyplex^®^ assays can be used to identify SARS-CoV-2 in patients with acute respiratory symptoms. In contrast to Liat^®^, a positive eazyplex^®^ result does not require confirmation due to the high specificity of the assay, as previously demonstrated. [6]. On the other hand, eazyplex^®^-negative samples need to be subsequently tested using RT-PCR. Depending on the actual prevalence, such a workflow may result in a significant additional workload and cost. However, it can be assumed that patients with acute respiratory illness in the ED who require hospitalization will generally be further tested using multiplex RT-PCR for different respiratory pathogens if the SARS-CoV-2 test is negative. When screening asymptomatic patients, negative Liat^®^ results almost rules out infection, but there is a significant rate of false-positive results, leading to an additional workload for verification to avoid unnecessary isolation of the patient. Use of the eazyplex^®^ assay cannot rule out infection, but it can identify those patients most likely to be infectious. The reduced sensitivity of a diagnostic assay can be problematic if the patient is at a pre-symptomatic stage [23]. Therefore, when RT-LAMP is used to screen asymptomatic patients, retesting for SARS-CoV-2 by RT-PCR must always be included in the differential diagnosis if the patient develops symptoms after hospital admission. This is also essential in cases where a patient who tested positive with Liat^®^ but whose positive result was not confirmed by RT-PCR becomes symptomatic.

**Figure 1:**
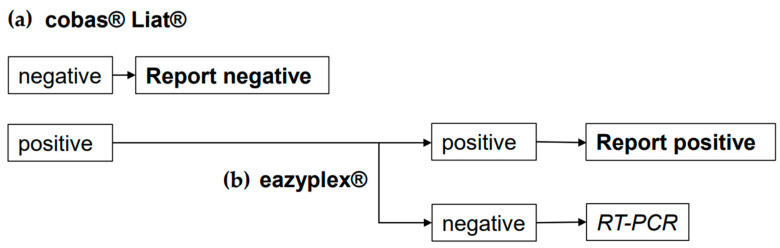
Proposed workflow combining the (**a**) cobas^®^ Liat^®^ and (**b**) eazyplex^®^ SARS-CoV-2 assays for rapid diagnosis of SARS-CoV-2 infection.

In conclusion, both rapid molecular assays are useful tools for the diagnosis of acute SARS-CoV-2 infection in high-priority patients. Figure 1 summarizes a diagnostic workflow that could be proposed when both assays are combined for rapid diagnosis of SARS-CoV-2 infection, regardless of the patient’s symptoms. An initial screening using Liat^®^ rules out infection if the test result is negative. Positive Liat^®^ results are tested via eazyplex^®^. A positive eazyplex^®^ result confirms that the patient is infected and infectious, and does not require further testing via RT-PCR. Their ease of use and short turnaround time allows both assays to be performed directly in the emergency department or in a satellite laboratory in the field; only specimens with a positive Liat result and a negative eazyplex^®^ result need to be retested via RT-PCR.

## Figures and Tables

**Table 1:** Confirmatory testing of positive cobas^®^ Liat^®^ SARS-CoV-2 results via RT-PCR.

Patient Group	cobas^®^ Liat^®^-Positive Samples (n)	Confirmatory Test		False-Positive Rate of cobas^®^ Liat^®^ (%)
		Positive	Negative	
Asymptomatic	157	115 ^a^	42	26.8
Symptomatic	221	213 ^a^	8	3.8

^a^ positive via RT-PCR and/or RT-LAMP.

**Table 2:** Positive percent agreement (PPA, sensitivity) of eazyplex^®^ RT-LAMP compared to reference RT-PCR.

Patient Group	RT-PCR Positive	eazyplex^®^ RT-LAMP		PPA, % (CI ^a^)
		Positive	Negative	
Asymptomatic	113	70	43	62 (52.3–70.9)
Symptomatic	212	192	20	90.6 (85.8–94.1)

^a^ CI, 95% confidence interval.

**Table 3:** Testing of positive cobas^®^ Liat^®^ SARS-CoV-2 samples using eazyplex^®^ RT-LAMP with regard to Ct value subgroups of confirmatory RT-PCR results.

Patient Group	Ct	Sample Number (% of All Positive Samples)	eazyplex^®^ RT-LAMP- Positive Results (%)
Asymptomatic	≤25	42 (37.1)	40 (95.2)
	25 < Ct < 30	28 (24.8)	24 (85.7)
	≥30	43 (38.1)	5 (11.7)
Symptomatic	≤25	150 (70.8)	150 (100)
	25 < Ct < 30	51 (24)	42 (82.4)
	≥30	11 (5.2)	1 (9)

**Table 4:** Comparison of cobas^®^ Liat^®^, eazyplex^®^, NeuMoDx™, and cobas^®^ SARS-CoV-2 assay results using INSTAND EQA ^a^ samples.

EQA Sample Number	Copies/mL ^b^	cobas^®^ Liat^®^	eazyplex^®^		NeuMoDx™		cobas^®^	
		Qualitative Result	Threshold Time, Min		Ct Value		Ct Value	
			N Gene	ORF8 Gene	N Gene	Nsp2 Gene	ORF1ab Gene	E Gene
340,094 ^c^	1.14 × 10^5^	positive	15.5	22.25	27.8	28.5	28.6	28.9
340,099 ^d^	1.11 × 10^5^	positive	13.5	-	27.2	27.8	28.5	29

^a^ INSTAND EQA, external quality assessment, June 2022. ^b^ Samples were provided as heat-inactivated virus isolates. ^c^ Omicron BA.2 (B.1.1.529). ^d^ Beta (B.1.351).

## Data Availability

The dataset analyzed in this study is available from the corresponding author upon reasonable request.
